# A phase III trial of topotecan and whole brain radiation therapy for patients with CNS-metastases due to lung cancer

**DOI:** 10.1038/sj.bjc.6604835

**Published:** 2009-01-06

**Authors:** T Neuhaus, Y Ko, R P Muller, G G Grabenbauer, J P Hedde, H Schueller, M Kocher, S Stier, R Fietkau

**Affiliations:** 1 Johanniter-Krankenhaus, Bonn, Germany; 2 Klinik für Strahlentherapie, Uniklinikum Köln, Köln, Germany; 3 Klinik für Strahlentherapie, Uniklinikum Erlangen, Erlangen, Germany; 4 Städt. Krankenhaus, Köln-Merheim, Germany; 5 Klinik für Strahlentherapie, Uniklinikum Bonn, Bonn, Germany; 6 Med. Poliklinik, Uniklinikum Bonn, Bonn, Germany; 7 Klinik für Strahlentherapie, Uniklinikum Rostock, Rostock, Germany

**Keywords:** brain metastases, lung cancer, radiochemotherapy, topotecan

## Abstract

Brain metastases represent an important cause of morbidity in patients with lung cancer and are associated with a mean survival of less than 6 months. Thus, new regimens improving the outcome of these patients are urgently needed. On the basis of promising data raised in a phase I/II trial, we initiated an open, randomised, prospective, multicentric phase III trial, comparing whole brain radiation therapy (WBRT; 20 × 2 Gy) alone with WBRT+topotecan (RCT; 0.4 mg m^−2^ day^−1^ × 20). A total of 320 patients with CNS-metastases due to SCLC or NSCLC were projected. The primary end point was overall survival, whereas second end points were local response and progression-free survival. However, until the cutoff date of study completion (i.e., a study duration of 34 months), only a total of 96 (RCT:47, WBRT:49) patients had been recruited, and so an analysis was performed at that time point. Although the numbers of grade 3/4 non-haematological toxicities (besides alopecia 115 (RCT/WBRT: 55 out of 60) were evenly distributed, the 25 haematological events occurred mainly in the combined treatment arm (24 out of 1). Local response, evaluated 2 weeks after treatment, was assessable in 44 (RCT/WBRT: 23 out of 21) patients, showing CR in eight (3 out of 5), PR in 17 (11 out of 6), SD in 14 (8 out of 6) and PD in five (1 out of 4) patients (all differences n.s.). Neither OAS (RCT/WBRT: median (days)): 87 out of 95, range 3–752/4–433; HR 1.32; 95% CI (0.83; 2.10)) nor PFS (median (days)): 71 out of 66, range, 3–399/4–228; HR 1.28, 95% CI (0.73; 2.43) differed significantly. On the basis of these results and the slow recruitment, a continuation of the study did not seem reasonable. The available data show no significant advantage for concurrent radiochemotherapy for patients with lung cancer; however, the recruited number of patients is too low to exhibit a small advantage of combined treatment.

Brain metastases represent an important cause of morbidity in patients with solid tumours. Most often, they were found in cancers of the lung with incidences in autoptic series of 54% in NSCLC and 80% in SCLC (Riva *et al*, 2001), and these tumour types account for more than one half of all brain metastases (Zimm *et al*, 1981).

Besides the local neurological complications, the occurrence of brain metastases is also a sign of systemic failure of tumour control, going ahead with a median survival time in untreated patients of about 1 month (Sundstrom *et al*, 1998). Thus, the treatment of brain metastases should focus on both, the control of neurological symptoms as well as a prolonged survival rate.

Although in patients with a limited number of CNS-metastases, surgery or stereotactic radiosurgery represents important therapeutic approaches (Patchell *et al*, 1990; Andrews *et al*, 2004; Jawahar *et al*, 2004), in those patients with multiple CNS-metastases, Whole Brain Radiotherapy (WBRT) is still the treatment of choice. A reduction of neurological complications is mostly achieved however, it results only in an overall survival benefit of approximately 4 months (Lagerwaard *et al*, 1999; Postmus, 1995; Saito *et al*, 2006), and a control of brain metastases will be found in only 50% of the patients (Lassman and DeAngelis, 2003).

As an alternative, the efficacy of chemotherapy in the treatment of brain metastases has been investigated. However, the response rates found in the treatment of brain metastases in mainly non-randomised studies were about 20–40% with a median survival time varying from 3 to 10 months and have thus been disappointing (Nieder *et al*, 2006).

Therefore, an improvement of these results is urgently needed. One approach was the addition of radiosensitisers to WBRT. However, the few studies on this subject published to date failed to show any significant effect on overall survival or on local response rate (Tsao *et al*, 2006).

For multimodal treatment strategies, including combination of chemotherapy (e.g., temozolomide, paclitaxel, nimustine, tegafur) with WBRT, sufficient data are rare, too. Although most of the studies pointed out an improvement in local control compared with WBRT, the median survival times did not differ (Postmus *et al*, 2000; Robinet *et al*, 2001; Ushio *et al*, 1991 Mornex *et al*, 2003).

A promising candidate for a combined radiochemotherapy seems to be topotecan, a topoisomerase I inhibitor with a well established activity in the systemic treatment of SCLC (von Pawel *et al*, 1999) and NSCLC (Stewart, 2004), a high brain capillary permeability (Sung *et al*, 1994; Baker *et al*, 1996) and radiosensitising effects (Kim *et al*, 1992; Lamond *et al*, 1996). For topotecan in a monotherapeutic setting, response rates of 33–63% in patients with brain metastases due to SCLC were found (Ardizzoni *et al*, 1997; Schutte *et al*, 1999; Korfel *et al*, 2002; summarised in Wong and Berkenblit, 2004).

As a consequence, three phase I/II-studies were initiated to evaluate the feasibility of topotecan-based combined radiochemotherapy in patients with brain metastases (Gruschow *et al*, 2002; Kocher *et al*, 2005; Hedde *et al*, 2007). In the setting of the dose-escalating phase I studies, Gruschow *et al* (2002) and Hedde *et al* (2007) found tolerable toxicities in those patients receiving 0.4 mg m^−2^ day^−1^ topotecan, either as a bolus or continuous infusion, concurrent with the WBRT (fraction size of 2 Gy day^−1^ for a total of 40 Gy). Kocher *et al* (2005) used a regimen with local target dosage of 36 Gy in 3-Gy-fractions and topotecan administered 12 × 0.5 mg m^−2^ day^−1^.

In the study published by Hedde *et al* (2007) 19% of their 68 patients developed grade 3/4 haematological and 21% developed grade 3/4 non-haematological events. Of their 47 patients, in whom a local response was assessable, seven presented with complete and 27 with partial responses (i.e. an overall response rate of 72%). The median survival of all patients reached 17 weeks and amounted to 33 weeks in responders.

Gruschow *et al* (2002) included 20 patients and found a median survival of 5 months. In 13 patients, the remission status could be evaluated; four complete and two partial responders were detected, resulting in an overall response rate of 46%.

Of the 47 patients who took part in the study of Kocher *et al* (2005), response evaluation was possible in 26 patients. In them, an overall response rate of 58% was described (5 out of 26 complete response (CR) and 10 out of 26 partial response (PR)). Grade 3/4 haematological toxicities were found in 25% of all patients.

On the basis of these promising results, we initiated a phase III study comparing the efficacy of a combined radiochemotherapy with topotecan and WBRT *vs* WBRT alone in patients with brain metastases from lung cancer.

## Patients and methods

### Patient eligibility

Patients with histologically proven lung cancer and intracerebral metastases have been entered in this open-label, prospective, multicentric, randomised phase III study. Initially only patients with recurrence of lung cancer after first line therapy could be included in the study. However, due to a slow recruitment, after 1 year an amendment allowing the inclusion of primary diagnosed patients was added. Patients were enrolled with ages between 18–75 years, and at least one measurable lesion in the brain was confirmed by computed tomography (CT) or magnetic resonance imaging. Sufficient bone marrow reserve was defined as neutrophil counts ⩾1500 *μ*l^−1^, leukocyte counts ⩾3500 *μ*l^−1^, platelet counts ⩾100 000 *μ*l^−1^ and haemoglobin ⩾9 g dl^−1^. Adequate renal function was defined by serum creatinine concentration ⩽1.5 mg% or creatinine clearance >60 ml min^−1^. Patients had to have a performance status of 0–2 according to ECOG criteria.

Exclusion criteria were prior to cerebral radiotherapy and/or surgery of cerebral metastases (except stereotactic biopsy), missing histologically proven nature of cancer, solitary intracerebral metastases suitable for neurosurgery, meningeosis carcinomatosa, active uncontrolled infection, concomitant or previous malignancies, except basal or squamous cell carcinoma or carcinoma *in situ* of the cervix and history of therapy with and/or known allergy to topoisomerase I inhibitors, pregnant or breast-feeding women. All patients were informed of the investigational nature of the study and had to provide written informed consent. The study was approved by local ethics committees of all participating centres.

Randomisation was performed by considering the parameters SCLC, NSCLC, extracerebral metastases and a number of brain metastases.

### Treatment protocol

Arm A (radiochemotherapy): Topotecan was administered as a 30 min infusion with 0.4 mg m^−2^ day^−1^ for 5 days over 4 weeks within 2 h before radiation therapy. Whole Brain Radiation (WBR) was applied with a fraction size of 2 Gy day^−1^ to a total of 40 Gy. Arm B (Radiotherapy): WBR was applied with a fraction size of 2 Gy day^−1^ to a total of 40 Gy.

Continuation therapy: subsequently, patients with extracerebral cancer lesions from both arms had the option to receive three additional cycles of topotecan chemotherapy (1.25 mg m^−2^ day^−1^, d1–5, q21d), starting on day 15 after the end of WBRT. In case a patient had not received any kind of chemo- or radiochemotherapy before entering the study, the institutionally preferred chemotherapeutic regimen was allowed to be used instead Continuation therapy was stopped after three cycles or when tumour progression of the extracerebral metastases occurred.

### Dose delays and modifications

In the case of severe neutropenia (neutrophil counts ⩽1000 *μ*l^−1^) or thrombocytopenia (platelet counts ⩽50 000 *μ*l^−1^), topotecan therapy was stopped until recovery of neutrophil counts to ⩾15 000 *μ*l^−1^ and of platelet counts to ⩾100 000 *μ*l^−1^. Radiation therapy was continued as planned. Therapeutic use of G-CSF was allowed and left to the decision of the treating physician. Antiemetic and supportive symptom treatment was left to the treating physician as well. For dexamethasone, a dosage of 4 mg two times per day was recommended; the dosage should not exceed 12 mg day^−1^. During the continuation therapy, a dose reduction of 0.25 mg m^−2^ day^−1^ topotecan for the next cycle was recommended in the case of thrombocytopenia (platelet counts ⩽50 000 *μ*l^−1^), whereas in the case of neutropenia, G-CSF should be applicated without reduction of the topotecan dosage. Again, therapy was continued after neutrophil counts increased to ⩾15 000 *μ*l^−1^ and platelet counts to ⩾100 000 *μ*l^−1^.

### Withdrawal from the study

The treatment was stopped as per the patients' wish, by the decision of the physician, tumour progression, severe side effects according to the NCIC CTCG guidelines or non-compliance of the patient. The whole study was to be stopped in case new therapeutic regimens with superior benefit of either therapy arm were published, if the interim analyses showed that the criteria for stopping the study by using the methods of Pocock (1978) and O'Brien and Fleming (1979) were reached and when the number of patients recruited was clearly below the expected value.

### Tumour assessment

Criteria for efficacy assessment were response rate, progression-free survival and overall survival. A complete response was defined as a complete disappearance of all evidence of disease in the brain. A partial response was defined as radiological response >50% in all brain metastases. Responses in tumour lesions <50% or increase in size less than 25% was defined as stable disease. A progressive disease was defined as either the occurrence of new lesions or an increase in size of more than 25%. Tumour assessment was performed with the method used initially. In addition, death caused by cerebral lesions was assessed if one of the following causes of death were described: Cerebral oedema, neurologic disorders, cramps, dementia and progression of cerebral lesions.

Tumour assessment was also performed for extracerebral lesions. Assessment of brain metastases by imaging was performed on day 15 after the end of radiotherapy/radiochemotherapy; in the case of a complete remission, it was repeated 4 weeks later. The final examination was planned for week 17 for patients with extracerebral tumours, and it included extracranial tumour imaging.

### Safety assessment

Baseline evaluations included medical history, physical examination, radiologic assessment of tumour status by cerebral CT or MR, conventional X-ray or CT of the lung and CT of the upper abdomen, ECOG scale, and laboratory evaluations. Full blood counts and biochemistry were measured weekly. On week 7 (15 days after end of the treatment), the following assessments were performed: physical examination, documentation of side effects, cerebral tumour status, neurologic status, blood counts and blood chemistry. During the continuation therapy full blood counts and biochemistry were measured before each course, and side effects were documented. On week 17, the final examination for all patients included physical examination, extracranial tumour imaging in the case of extracerebral tumours, neurologic status, laboratory evaluations and documentation of palliative treatment. During follow-up visits, the toxicities and palliative treatments were documented every 8 weeks.

### Quality of life

For recording the quality of life we used the EORTC-QLQ C30A questionnaire added by the brain cancer module EORTC-QLQ BN20. The patients should get these questionnaires before treatment, 2 weeks after treatment and at follow-up. However, an analysis was not possible owing to the very low number of returned questionnaires.

### Statistical considerations

The primary end point was overall survival (OAS). Secondary end points were progression-free survival, rates of complete responses of the cerebral lesions, duration of remission, status of the extracerebral tumours after continuation therapy and toxicity.

As mentioned, the primary end point with regard to therapeutic efficacy is the survival time of the patients starting at the randomisation time point. Event is the time of death. The sample size estimate for the study was based on a sequential study design with two samples and an interim analysis. The reference value was the median survival time of 4 months under standard therapy (radiotherapy, Arm B). The estimated minimum clinically relevant therapeutic effect under investigational treatment (radiochemotherapy, Arm A) was an increase in the survival time in the interventional group to 5.5 months. The study protocol justifies the one-sided testing by the fact that a longer survival time in patients under the investigational therapy can be assumed to be highly likely. For the testing of this study design, the estimated number of patients was 320 (i.e., 160 patients per treatment arm with equal distribution). Dropouts were not considered.

An interim analysis was planned after the death of the first 150 patients (event of primary end point); the decision concerning the continuation of the study was based on using the *α*-spending principle with criteria for closing the study described by Pocock (1978) and O'Brien and Fleming, (1979).

Overall survival was defined as the interval from randomisation until death. Progression-free survival was defined as the interval from randomisation until evidence of disease progression or death. Analysis was performed by the Kaplan–Meier estimation. Overall survival and progression-free survival were compared using the log-rank test. Regression analyses were performed by using the Cox proportional hazards model. The analyses of all other secondary end points were evaluated in an explorative or descriptive manner.

## Results

### Patient characteristics

The first patient was included in October 2001. An interim analysis was planned after the death of 150 patients. However, until August 6, 2004, that is, after a study duration of 34 months, only 95 patients in 11 centres had been recruited, and so the interim analysis was performed at that time point. This analysis did not show any benefit of radiochemotherapy with regard to overall survival and thus, on the basis of the slow recruitment and the result of the interim analysis, a continuation of the study did no longer appear reasonable. The results described here represent the final analysis, in which 96 patients were included.

The demographic data as presented in Table 1 were evenly distributed in the two groups. Most of the patients (64%) had a good ECOG performance status of 0–1, and 75% of the patients had extracerebral metastases. There were 25 patients (26%) with more than four CNS-metastases, and 15.6% of the patients received continuation therapy with topotecan.

### Safety and tolerability

Only half of the patients (51%) were reported to be treated per protocol, whereas 49% of the patients were not. The reasons for protocol deviations are mainly early deaths, haematological toxicities, dosage failure, worsening of general condition and tumour progression. In detail, in arm A the chemotherapy was delayed or reduced in nine patients because of neutropenia, and in six of them G-CSF was given at least once. Although no patient stopped topotecan because of neutropenia, one patient left the study because of prolonged thrombocytopenia.

The main cause of early death (Table 2), defined as death within 6 weeks after recruitment, was tumour progression. Early death occurred in 24% of the patients, and of these 61% belonged to the arm receiving radiochemotherapy. The main causes of death in general were tumour progression, especially in 18 patients' (3 arm A, 15 arm B) progression of cerebral lesions.

The occurrence of non-haematological grade 3/4 toxicities did not differ between both treatment arms (Table 3). In total, besides alopecia found in about 40% of all patients, 118 non-haematotoxic grade 3/4 adverse events were described. Haematological toxicities occurred – as expected – more often in the radiochemotherapy arm (Table 3). All in all, 25 grade 3/4 haematotoxic adverse events were reported, 24 in the patients receiving the combined approach and one in the patient treated with WBRT alone.

### Response

Response of brain metastases, evaluated about 2 weeks after treatment, was assessable in 44 patients (radiochemotherapy/radiotherapy: 23 out of 21), showing CR in eight (3 out of 5), PR in 17 (11 out of 6), stable disease (SD) in 14 (8 out of 6) and progressive disease (PD) in five (1 out of 4) patients (Table 4). Data concerning the response behaviour of extracerebral metastases after continuation therapy were available in just 10 (radiochemotherapy/radiotherapy: 6 out of 4) patients. Although in the patients receiving radiochemotherapy 2 PR, 1 SD and 3 PD were found, all four patients in the radiotherapy-arm presented with PD.

**Figure 1:**
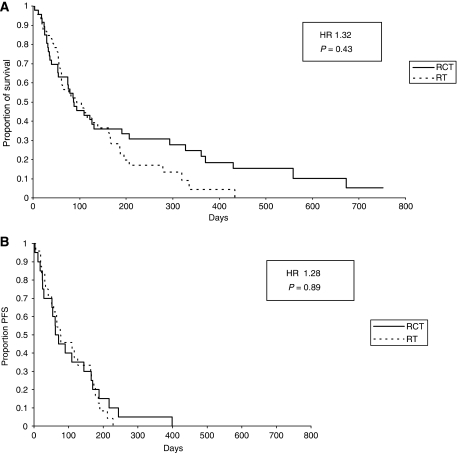
Overall survival (**A**) and progression-free survival (**B**) in both arms (HR=Hazard ratio; RCT=radiochemotherapy; RT=radiotherapy).

Neither overall survival (radiochemotherapy/radiotherapy: median (days): 87 out of 95, range 3–752 out of 4–433; Hazard Ratio 1.32, 95% CI 0.83–2.1) nor progression-free survival (assessable in 44 patients; median (days): 71 out of 66, range: 3–399 out of 4–228; Hazard Ratio 1.28, 95% CI 0.73–2.43) differed significantly between the two groups (using Cox–Mantel test; Figure 1A and B). This was true for SCLC and NSCLC, respectively, and thus the histology of the lung cancer did not seem to influence the response rates.

The co-medication was not analysed in detail, but although serum concentration of topotecan could be diminished in patients treated with inducers of CYP450-enzymes, the investigators negotiated any relevant influence of these inducers on the results of the study.

## Discussion

The main goal of the presented study was to elucidate the relevance of concurrent radiochemotherapy with topotecan (0.4 mg m^−2^ day^−1^ for 5 days over 4 weeks) and WBRT (2 Gy day^−1^, 40 Gy in total) in patients with brain metastases due to lung cancer in comparison with WBRT alone concerning overall survival. We have chosen a schedule of a 2-Gy fraction because we wanted to use the radiosensitising effect of topotecan; thus, both WBRT and chemotherapy should be administered at the same time and over the same period. Second, there are no data concerning higher doses of radiotherapy together with topotecan; the toxicity may increase in an uncalculated manner when 4 or 5 Gy are used. On the other hand, a treatment course of 4 weeks is a long period for patients with a short life expectancy, as is true in patients with brain metastases. Thus, a shortened schedule would be more attractive for both patients and physicians, and this should be kept in mind when planning such trials.

To test this hypothesis, 320 patients needed to be enrolled. The expected recruitment period lasted 36 months. However, after 34 months, just 96 patients could be included in the study. At this time, an interim analysis was performed that led to the results described above. The low recruitment was mainly caused by patients with a limited number of cerebral metastases and by the resulting opportunity of a local treatment (surgery or stereotactic radiosurgery). As the recruitment rates differed to a greater amount between the different centres, we assume that in addition some centres overestimated the numbers of patients they were able to include. The data presented here once again show the difficulties in estimating the numbers of patients that may be included in each centre, especially in trials with a wide range of exclusion criteria. To avoid such problems, either the number of participating centres should be enlarged or the centres chosen must meet certain requirements.

In the study presented here, the overall response rate in the patients receiving radiochemotherapy was 60%, whereas it was 52% in the WBRT arm. Compared with our own previous data generated in a phase I/II study, where we found overall response rates of 72% (Hedde *et al*, 2007), the outcome of the patients treated with a combined approach was inferior. However, our results confirm the findings of the only two comparable studies testing a topotecan-based radiochemotherapy, in which overall response rates of 46 and 58%, respectively, were described (Gruschow *et al*, 2002; Kocher *et al*, 2005).

With regard to the observed response rates, it was not surprising that neither OAS nor PFS were increased in the combined treatment arm. This was true independent of the kind of lung cancer; thus, even in patients with SCLC, possibly because of the low numbers of patients in each subgroup, we were not able to show improved results when using the combined approach. As lung cancer is not a homogeneous disease, further studies of such a kind should focus on either NSCLC or SCLC to get the definite answer that was missed here. In addition, this approach would have the advantage of being able to choose a chemotherapy optimal for the respective cancer.

Besides the response, another interesting point in brain metastases is the diminished quality of life, which should be observed and analysed in trials dealing with this disease for answering the question whether more aggressive treatment is feasible regarding quality of life. However, our study failed to answer this question owing to a very low return of the questionnaires. In further studies, the questions should be asked in a personal meeting or telephone call by a certain member of the team instead of just handing over the questionnaire.

The results presented in this study are in line with data from the very few randomised studies, with a total of only 264 participants comparing radiochemotherapy (by using temozolomide, carboplatin or methyl-CCNU) with radiotherapy alone in patients with brain metastases of solid tumours (Ushio *et al*, 1991; Antonadou *et al*, 2002; Guerrieri *et al*, 2004; Verger *et al*, 2005), as neither of these found an influence of the overall survival. In two of these studies (Ushio *et al*, 1991; Antonadou *et al*, 2002), the objective response rates (96 *vs* 67% and 74 *vs* 36%, respectively) were significantly improved in the patients receiving combined treatment. However, the response rates mentioned there were remarkably high.

Two other studies compared the efficacy of chemotherapy alone with a combination with WBRT (Postmus *et al*, 2000; Robinet *et al*, 2001). Postmus *et al* (2000) used teniposide in 120 patients with SCLC, and the combined approach resulted in elevated response rates (57 *vs* 22%) and a significantly prolonged time-to-progression (6.5 *vs* 4 months). Robinet *et al* (2001), by using cisplatin/vinorelbine with or without WBRT, found response rates of 33 and 27%, respectively. Again, the OAS was not influenced by either study.

In summary, on the basis of the results presented here and the slow recruitment, a continuation of the study did not seem reasonable. The available data of the only phase III trial concerning a combined treatment with topotecan and WBRT in patients with brain metastases due to lung cancer show no advantage for concurrent radiochemotherapy; however, the recruited number of patients is too low to exhibit a small advantage of combined treatment.

## Figures and Tables

**Table 1:** Demographic results

	**Radiochemotherapy**	**Radiotherapy**
No. of patients	47	49
		
*Age*		
Median	58	59
Range	34–75	42–75
Male/female	32/15	30/19
		
*Primary site of tumour*		
NSCLC first line	7	8
NSCLC recurrence	24	24
SCLC first line	3	2
SCLC recurrence	13	15
		
*ECOG performance status*		
0	8	2
1	29	22
2	7	19
3	1	2
Not reported	2	4
		
*No. of brain metastases >4*		
Yes	11	14
No	36	35
		
*Extracerebral metastases*		
Yes	35	37
No	12	12
		
*Dexamethasone dosing*		
Yes	38	39
Not	9	8
Not reported	0	2
		
*Courses of continuation therapy*		
1	1	2
2	1	1
3	6	4

**Table 2:** Causes of early death (death within 6 weeks after recruitment)

	**Radiochemotherapy**	**Radiotherapy**
Early death	14/47	9/49
Tumour progression	7	6
Cardiovascular failure	2	0
Pulmonary embolism	1	1
Infection	2	1
GI-haemorrhage	2	0
Unknown	0	1

**Table 3:** Incidence of the main grade 3 and 4 adverse events (by patients)

	**Radiochemotherapy**				**Radiotherapy**			
	**Grade 3**		**Grade 4**		**Grade 3**		**Grade 4**	
**CTC-Grade**	**No.of patients**	**Percentage of patients**	**No.of patients**	**Percentage of patients**	**No.of patients**	**Percentage of patients**	**No.of patients**	**Percentage of patients**
Granulocytes	1	2.1	3	6.4	1	2.0	0	0.0
Haemoglobin	2	4.3	0	0.0	0	0.0	0	0.0
Leukocytes	5	10.6	2	4.3	0	0.0	0	0.0
Thrombocytes	8	17.1	3	6.4	0	0.0	0	0.0
Alopecia	20	42.6	0	0.0	19	38.8	0	0.0
Infection	8	17.0	4	8.5	5	10.2	1	2.0
Somnolence	2	4.3	0	0.0	3	6.1	0	0.0
Dyspnea	4	8.5	1	2.1	0	0.0	1	2.0
Nausea	4	8.5	0	0.0	3	6.1	0	0.0
Vomiting	1	2.1	0	0.0	0	0.0	0	0.0
Constipation	3	6.4	2	4.3	3	6.1	0	0.0
Pain	5	10.6	2	4.3	7	14.3	5	10.2
Stomatitis	3	6.4	0	0.0	5	10.2	1	2.0
Hyperglycaemia	2	4.3	0	0.0	1	2.0	0	0.0
GI-haemorrhage	1	2.1	0	0.0	1	2.0	0	0.0
Sensorium	2	4.3	1	2.1	1	2.0	0	0.0
Creatinin-elevated	2	4.3	0	0.0	3	6.1	0	0.0
Pneumonitis	0	0.0	1	2.1	3	6.1	0	0.0

**Table 4:** Response of brain metastases

	**Radiochemotherapy**	**Radiotherapy**
	**47**	**49**
Complete response (CR)	3	5
Partial response (PR)	11	6
Stable disease (SD)	8	6
Progressive disease (PD)	1	4
Not specified	2	3
Not assessable	22	25
